# Primary Care implementation of Germ Defence, a digital behaviour change intervention to improve household infection control during the COVID-19 pandemic: A structured summary of a study protocol for a randomised controlled trial

**DOI:** 10.1186/s13063-021-05188-7

**Published:** 2021-04-09

**Authors:** Jeremy Horwood, Melanie Chalder, Ben Ainsworth, James Denison-Day, Frank de Vocht, Martha M. C. Elwenspoek, Pippa Craggs, Rachel Denholm, Jonathan Sterne, Cathy Rice, Sascha Miller, Beth Stuart, Paul Little, Michael Moore, Merlin Willcox, John Macleod, Martin Gullford, Kate Morton, Lauren Towler, Nick Francis, Richard Amlôt, Lucy Yardley

**Affiliations:** 1 National Institute for Health Research, Applied Research Collaboration West (NIHR ARC West) University Hospitals Bristol and Weston NHS Foundation Trust, 9th Floor, Whitefriars, Lewins Mead, Bristol, BS1 2NT UK; 2 Centre for Academic Primary Care (CAPC), Bristol Medical School, Population Health Sciences, University of Bristol, Canynge Hall, 39 Whatley Road, Bristol, BS8 2PS UK; 3 NIHR Health Protection Research Unit (HPRU) in Behavioural Science and Evaluation, University of Bristol, Bristol, UK; 4 Department of Psychology, University of Bath, Bath, UK; 5 School of Psychology, University of Southampton, Southampton, UK; 6 Stoke Gifford, UK; 7 Faculty of Medicine, University of Southampton, Southampton, UK; 8 School of Population Health and Environmental Sciences at King’s College London, London, UK; 9 Behavioural Science Team, Emergency Response Department, Public Health England, Salisbury, UK; 10 School of Psychological Science, University of Bristol, Bristol, UK

**Keywords:** COVID-19, Randomised controlled trial, Protocol, Primary care, Behaviour change, Digital medicine, Infection control, Infectious disease, Protection, Digital health

## Abstract

**Objectives:**

To examine the effectiveness of randomising dissemination of the Germ Defence behaviour change website via GP practices across England UK.

**Trial design:**

A two-arm (1:1 ratio) cluster randomised controlled trial implementing Germ Defence via GP practices compared with usual care.

**Participants:**

Setting: All Primary care GP practices in England. Participants: All patients aged 16 years and over who were granted access by participating GP practices.

**Intervention and comparator:**

Intervention: We will ask staff at GP practices randomised to the intervention arm to share the weblink to Germ Defence with all adult patients registered at their practice during the 4-month trial implementation period and care will otherwise follow current standard management. Germ Defence is an interactive website (http://GermDefence.org/) employing behaviour change techniques and practical advice on how to reduce the spread of infection in the home. The coronavirus version of Germ Defence helps people understand what measures to take and when to take them to avoid infection. This includes hand washing, avoiding sharing rooms and surfaces, dealing with deliveries and ventilating rooms. Using behaviour change techniques, it helps users think through and adopt better home hygiene habits and find ways to solve any barriers, providing personalised goal setting and tailored advice that fits users’ personal circumstances and problem solving to overcome barriers.

Comparator: Patients at GP practices randomised to the usual care arm will receive current standard management for the 4-month trial period after which we will ask staff to share the link to Germ Defence with all adult patients registered at their practice.

**Main outcomes:**

The primary outcome is the effects of implementing Germ Defence on prevalence of all respiratory tract infection diagnoses during the 4-month trial implementation period.

The secondary outcomes are:

1) incidence of COVID-19 diagnoses

2) incidence of COVID-19 symptom presentation

3) incidence of gastrointestinal infections

4) number of primary care consultations

5) antibiotic usage

6) hospital admissions

7) uptake of GP practices disseminating Germ Defence to their patients

8) usage of the Germ Defence website by individuals who were granted access by their GP practice

**Randomisation:**

GP practices will be randomised on a 1:1 basis by the independent Bristol Randomised Trials Collaboration (BRTC). Clinical Commission Groups (CCGs) in England will be divided into blocks according to region, and equal numbers in each block will be randomly allocated to intervention or usual care. The randomisation schedule will be generated in Stata statistical software by a statistician not otherwise involved in the enrolment of general practices into the study.

**Blinding (masking):**

The principal investigators, the statistician and study collaborators will remain blinded from the identity of randomised practices until the end of the study.

**Numbers to be randomised (sample size):**

To detect planned effect size (based on PRIMIT trial, Little et al, 2015): 11.1 million respondents from 6822 active GP practices. Assuming 25% of these GP practices will engage, we will contact all GP practices in England spread across 135 Clinical Commissioning Groups.

**Trial status:**

Protocol version 2.0, dated 13 January 2021. Implementation is ongoing. The implementation period started on 10 November 2020 and will end on 10 March 2021.

**Trial registration:**

This trial was registered in the ISRCTN registry (isrctn.com/ISRCTN14602359) on 12 August 2020.

**Full protocol:**

The full protocol is attached as an additional file, accessible from the Trials website (Additional file 1). In the interest in expediting dissemination of this material, the familiar formatting has been eliminated; this Letter serves as a summary of the key elements of the full protocol.

**Supplementary Information:**

The online version contains supplementary material available at 10.1186/s13063-021-05188-7.

## Supplementary Information

**Additional file 1.** Full Study Protocol.

## Data Availability

Not applicable.

