# Supplementary material for: Primary Care implementation of Germ Defence, a digital behaviour change intervention to improve household infection control during the COVID-19 pandemic: A structured summary of a study protocol for a randomised controlled trial
Source: Trials. 2021 Apr 9;22:263. doi: 10.1186/s13063-021-05188-7 (PMC8033092; doi:10.1186/s13063-021-05188-7)
Supplement: Supplementary file 1 — Additional file 1. Full Study Protocol. [file 13063_2021_5188_MOESM1_ESM.pdf]

Full title: **Primary Care implementation of Germ Defence: A digital behaviour change intervention to improve infection control during the COVID-19 pandemic**

Public title: **Germ Defence: a website to improve infection control during the COVID-19 pandemic**

Short title: **COVID-19 Germ Defence Implementation**

**Protocol version number:**	v2.0
**Protocol date:**	13 January 2021
**Funder reference number:**	MC_PC_19068
**Study sponsor number:**	2020-3213
**IRAS project ID:**	287978
**ISRCTN registration:**	ISRCTN14602359
**REC reference:**	20/YH/0261

.

TABLE OF CONTENTS

I. KEY STUDY CONTACTS 1
II. LIST OF ABBREVIATIONS 2
III. STUDY SUMMARY 3
IV. STUDY MANAGEMENT 4
1. PLAIN ENGLISH SUMMARY 5
2. BACKGROUND 6
FIGURE 1: IMPLEMENTATION FLOW CHART 7
3. RATIONALE 7
4. RESEARCH AIM, OBJECTIVES AND OUTCOME MEASURES 8
4.1 AIM 8
4.2 OBJECTIVES 8
4.3 OUTCOME MEASURES 8
4.4 MEASUREMENT OF CLINICAL OUTCOMES 8
5. STUDY DESIGN & SETTING 9
5.1 STUDY DESIGN 9
5.2 INTERVENTION 9
5.3 STUDY SETTING 9
5.4 ELIGIBILITY CRITERIA 10
5.5 ROUTINELY COLLECTED DATA 10
5.6 STUDY DURATION 10
6. RECRUITMENT AND RANDOMISATION 11
6.1 RECRUITMENT 11
6.2 RANDOMISATION 11
7. STATISTICS AND DATA ANALYSIS 11
7.1 POWER CALCULATION 11
7.2 STATISTICAL ANALYSIS PLAN 12
8. DATA MANAGEMENT 13
8.1 GENERAL APPROACH 13
8.2 ARCHIVING 14
8.3 DATA SHARING 14
9. ETHICAL AND REGULATORY CONSIDERATIONS 14
9.1 RESEARCH APPROVALS 14
9.2 RISKS AND BENEFITS 14
9.3 STUDY SPONSORSHIP 14
9.4 INVESTIGATORS' RESPONSIBILITIES 14
9.5 CONSENT 15
9.6 CONFIDENTIALITY 15
9.7 PUBLIC AND PATIENT INVOLVEMENT 15
9.8 INDEMNITY 15
10. DISSEMINATION 15
11. REFERENCES 16
APPENDIX 1: STUDY PROJECT PLAN 17

I. KEY STUDY CONTACTS

Chief Investigator	Dr Jeremy Horwood, Associate Professor of Social Sciences and Health, NIHR ARC West/NIHR HPRU Behavioural Science and Evaluation/Centre for Academic Primary Care (CAPC), Bristol Medical School, University of Bristol (UoB) j.horwood@bristol.ac.uk
Sponsor	Adam Taylor, Head of Research Governance, Research and Enterprise Development, University of Bristol<br>(0)117 428 4092, adam.taylor@bristol.ac.uk
Funders	UKRI Coronavirus Rapid Response Call (MC_PC 19068) and National Institute for Health Research, Applied Research Collaboration West (NIHR ARC West) and NIHR Health Protection Research Unit (HPRU) in Behavioural Science and Evaluation

Key Protocol Contributors

Dr Ben Ainsworth, Lecture in Health Psychology, University of Bath. b.ainsworth@bath.ac.uk

Dr Richard Amlôt, Scientific Programme Leader of the Behavioural Science Team, Emergency Response Department, Public Health England. richard.amlot@phe.gov.uk

Dr Melanie Chalder, Research Fellow/Trial Manager, Bristol Medical School, UoB. melanie.chalder@bristol.ac.uk

Ms Pippa Craggs, Trial Manager, ARC West/Bristol Medical School, UoB. pippa.craggs@bristol.ac.uk

Dr Sarah Denford, Research Fellow, Bristol Medical School, UoB. sarah.denford@bristol.ac.uk

Dr Rachel Denholm, Lecturer in Applied Health Data Science, Bristol Medical School, UoB. r.denholm@bristol.ac.uk

Dr James Denison-Day, Research Fellow, University of Southampton. J.L.Denison-Day@soton.ac.uk

Dr Frank de Vocht, Reader in Epidemiology and Public Health, ARC West/Bristol Medical School, UoB. frank.devocht@bristol.ac.uk

Dr Martha Elwenspoek, Research Associate, ARC West/Bristol Medical School, UoB. martha.elwenspoek@bristol.ac.uk

Prof Martin Gullford, Professor of Public Health in the School of Population Health and Environmental Sciences at King's College London martin.gulliford@kcl.ac.uk

Dr Maya Gobin, Consultant Epidemiologist, Field Service, Public Health England. Maya.Gobin@phe.gov.uk

Prof Michael Moore, Professor of Primary Care Research, University of Southampton, mvm198@soton.ac.uk

Dr Merlin Willcox, Academic Clinical Lecturer in Primary Care, University of Southampton, M.L.Willcox@soton.ac.uk

Prof Nick Francis, Professor of Primary Care Research. School of Medicine University of Southampton, nick.francis@soton.ac.uk

Prof Paul Little, Professor of Primary Care Research, University of Southampton, P.Little@soton.ac.uk

Prof John Macleod, Professor in Clinical Epidemiology and Primary Health Care, NIHR ARC West/CAPC, Bristol Medical School, UoB. john.macleod@bristol.ac.uk

Ms Sascha Miller, Postgraduate Researcher, University of Southampton, S.J.Miller@soton.ac.uk

Prof Michael Moore, Professor of Primary Health Care Research, University of Southampton. mvm198@soton.ac.uk

Dr Kate Morton, Research Fellow, University of Southampton. K.S.Morton@soton.ac.uk

Ms Cathy Rice, Public contributor. Cathy.Rice@uwe.ac.uk

Prof Jonathan Sterne, Professor of Medical Statistics and Epidemiology, UoB. jonathan.sterne@bristol.ac.uk

Dr Beth Stuart, Associate Professor, University of Southampton. bls1@soton.ac.uk

Ms Lauren Towler, PhD student, University of Southampton. lbt1g14@soton.ac.uk

Dr Merlin Willcox, Academic Clinical Lecturer in Primary Care, University of Southampton. M.L.Willcox@soton.ac.uk

Prof Lucy Yardley, Professor of Health Psychology, NIHR ARC West/NIHR HPRU Behavioural Science and Evaluation/CAPC, UoB and University of Southampton. lucy.yardley@bristol.ac.uk

II. LIST OF ABBREVIATIONS

ARC	Applied Research Collaboration
BJGP	British Journal of General Practice
BJPH	British Journal of Public Health
BMJ	British Medical Journal
BRTC	Bristol Randomised Trials Collaborative
CAPC	Centre for Academic Primary Care
CCG	Clinical Commissioning Group
CCTV	closed-circuit television
CHESS	COVID-19 Hospitalisation in England Surveillance System
CI	Chief Investigator
CRN	Clinical Research Networks
DARS	Data Access Request Service
DiD	difference-in-differences
DPA	Data Protection Act
FLUFF	Facility for the Upload of Large Files
GDPR	General Data Protection Regulation
GLMM	generalised linear mixed effects model
GP	general practitioner
GPES	General practice extraction service
HDRUK	Health Data Research United Kingdom
HES	Hospital Episode Statistics
HPRU	Health Protection Research Unit
HRA	Health Research Authority
IT	information technology
ITT	intention-to-treat
MRC	Medical Research Council
NHS	National Health Service
NIHR	National Institute of Health Research
OID	Organisation Information Document
ONS	Office of National Statistics
PIC	Participant Identification Centre
PMG	Project Management Group
PPI	patient and public involvement
RCGP	Royal College of General Practitioners
RDSF	Research Data Storage Facility
REC	Research Ethics Committee
RTI	respiratory tract infection

SAPC	Society for Academic Primary Care
SOP	standard operating procedure
TMF	trial management file
UK	United Kingdom
UKRI	United Kingdom Research and Innovation
UKSBM	United Kingdom Society for Behavioural Medicine
UoB	University of Bristol

III. STUDY SUMMARY

Study title	Primary Care implementation of Germ Defence: A digital behaviour change intervention to improve infection control during the COVID-19 pandemic	
Public title	Germ Defence: a website to improve infection control during the COVID-19 pandemic	
Short title	COVID-19 Germ Defence Implementation	
Study design	Two-arm cluster randomised trial implementing Germ Defence via GP practices compared with usual care	
Intervention	Germ Defence - digital behaviour change interactive website to improve infection control. Full details available at: http://Germ Defence.org/	
Study participants	GP practices in England	
Sample size	To detect planned effect size (based on PRIMIT trial, Little et al, 2015): 11.1 million respondents from 5,936 active GP practices. Assuming 25% of these GP practices will engage, we will contact all GP practices in England spread across 135 Clinical Commissioning Groups.	
Study period	1 October 2020 to 30 April 2021	
Outcomes	*Objectives*	*Outcome measures*
Primary	Examine effects of implementing Germ Defence on prevalence of all respiratory tract infection diagnoses	Routine data – OpenSAFELY Oxford University's secure analytics platform (https://opensafely.org/) or GPES [general practice extraction service] data for pandemic planning and research (COVID-19), and is collected by NHS Digital
Secondary	Incidence of COVID-19 diagnoses	Routine data - GPES or OpenSAFELY
	Incidence of COVID-19 symptom presentation	Routine data - GPES or OpenSAFELY
	Incidence of gastrointestinal infections	Routine data - GPES or OpenSAFELY
	Number of primary care consultations	Routine data - GPES or OpenSAFELY
	Antibiotic usage	Routine data - GPES or OpenSAFELY

	Hospital admissions	Routine data - GPES or OpenSAFELY
	Uptake of GP practices disseminating Germ Defence to their patients	Germ Defence website analytics
	Usage of the Germ Defence website by individuals who were granted access by their GP practice	Germ Defence website analytics

IV. STUDY MANAGEMENT

Sponsor
The Sponsor will be the University of Bristol who will ensure the study has adequate insurance and meets all regulatory obligations.

Funder
This research is funded by UKRI Coronavirus Rapid Response Call (CV220-009) and National Institute for Health Research, Applied Research Collaboration West (NIHR ARC West) and NIHR Health Protection Research Unit (HPRU) in Behavioural Science and Evaluation.

Project Management Group (PMG)
The study will be supervised by a PMG and will consist of Lucy Yardley, Ben Ainsworth, Richard Amlôt, Melanie Chalder, Pippa Craggs, Sarah Denford, Rachel Denholm, James Dennison-Day, Frank De Vocht, Martha Elwenspoek, Maya Gobin, Jeremy Horwood, Paul Little, John Macleod, Sascha Miller, Michael Moore, Kate Morton, Cathy Rice, Jonathan Sterne, Beth Stuart, Lauren Towler, Merlin Willcox. The PMG will meet every month via conference call.

Patient and Public Involvement (PPI)
Cathy Rice - who was a co-investigator on the original Germ Defence team and contributed to writing the funding proposal, updating and optimising the content of the intervention and co-authoring publications - continues to contribute to the Germ Defence work by commenting on this project's study design and protocol development. She will also be a member of the monthly Project Management Group who will discuss and guide study progress.

1. PLAIN ENGLISH SUMMARY

Project description

Recent research into coronavirus has shown that members of the public can play a crucial role in controlling infection outbreaks, by adopting simple behaviours to curb the spread of infection in the home such as handwashing, cleaning surfaces, wearing of face coverings and social distancing. Despite public health advice, evidence shows most people need to change their behaviour to help prevent infection.

Germ Defence is an interactive website (http://Germ Defence.org/) employing behaviour change techniques to supplement public health advice. Germ Defence was originally developed during the H1N1 swine flu pandemic using theory, evidence and extensive feedback from members of the public. It was then trialled in over 20,000 patients and shown to reduce the number and severity of infections of users and members of their household. Germ Defence has recently been updated for the COVID-19 pandemic. The website aims to help users with pre-planning about effective isolation of an infected household member; personalised goal setting for increasing a range of infection control behaviours; changing the home environment to support new habits and problem solving to overcome barriers.

What is the aim of the project?

This project aims to examine the effects of randomising dissemination of the Germ Defence website via GP practices on rates of respiratory infection, including COVID-19 and seasonal 'flu.

What are we doing?

We will contact every GP practice in England and ask them to share the link to Germ Defence with their patients. Half of the practices will be randomly chosen and asked to immediately send out the Germ Defence link to their patients in the Autumn of 2020. These practices will be known as the intervention arm or immediate implementation group. The other half of the practices will be contacted to send out Germ Defence in March 2021 and will be known as the usual care arm or delayed implementation group. We will assess usage of the Germ Defence link from anonymous data produced by the website. We will then use anonymised national data that is collected as part of routine care to compare whether infection rates are lower in practices that sent Germ Defence information to their patients immediately, compared to those that didn't send the information until later.

2. BACKGROUND

Germ Defence - a digital behaviour change intervention - was shown to increase handwashing and reduce respiratory tract infection in a randomised controlled trial of more than 20,000 households (Little et al., 2015). Germ Defence proved it can help reduce the risk of infection by around 14% (Little et al, 2015). The trial took place during the 2009/10 outbreak of swine 'flu, when there were already increased public health messages about reducing the spread of viruses through handwashing. Despite this raised awareness, people who used the website were still more likely to wash their hands the recommended amount of 10 times a day compared with those who did not. As a result, they caught fewer colds and 'flu, and recovered more quickly from these ailments. This reduction in illness also extended to the people who lived with them, leading to a lower demand for consultations with their doctors.

The intervention was developed using the person-based approach, which involved conducting in-depth qualitative research to develop a detailed understanding of how to overcome the behavioural barriers users might encounter to engaging with the target behaviours (Yardley et al., 2015). The intervention development drew on both theory and evidence, and incorporates education, personalised goal setting, environmental prompting and emotional motivation (Ainsworth et al., 2016). A process evaluation showed that the intervention was effective for both men and women, older and younger people, and for people with both lower and higher levels of education (Ainsworth et al., 2016). Importantly, the biggest change in handwashing behaviour occurred after the first of four sessions, so the intervention structure was adapted to comprise of one stand-alone session with further optional content available immediately afterwards (Ainsworth et al., 2016). The adapted intervention was disseminated 'in the wild' to help reduce seasonal colds and 'flu.

Germ Defence has recently been further optimised to respond to the need to reduce the spread of the COVID-19 virus. The intervention was modified in line with the latest guidance from the Medical Research Council (MRC) on intervention adaptation (Evans et al., 2019) and expert advice from clinicians, public health experts, health psychologists, behaviour change specialists and policy makers, alongside a range of patient and public involvement activities. In line with the person-based approach, in-depth qualitative interviews were conducted with a wide range of people about the Germ Defence website (Yardley et al., 2015). This enabled the research team to identify people's beliefs about each page, to understand possible barriers to enacting the target behaviours in a real-life setting, and to further optimise the intervention so it is as persuasive and effective as possible (Bradbury et al., 2018).

Germ Defence has been designed so that anyone can use and benefit from its information and ideas on how to lower their risk of catching COVID-19. This includes specific techniques for handwashing (when, where and how to wash hands effectively), keeping a safe distance, and not touching the face as well as information to help people decide if they need to wear face coverings and how to minimise their viral load (Little et al., 2020). In addition, the website provides advice on how people might look after family and friends who are ill, but still protect themselves. Rapid dissemination is now underway, and the Germ Defence website has already had more than 12,000 visits in the UK.

FIGURE 1: IMPLEMENTATION FLOW CHART

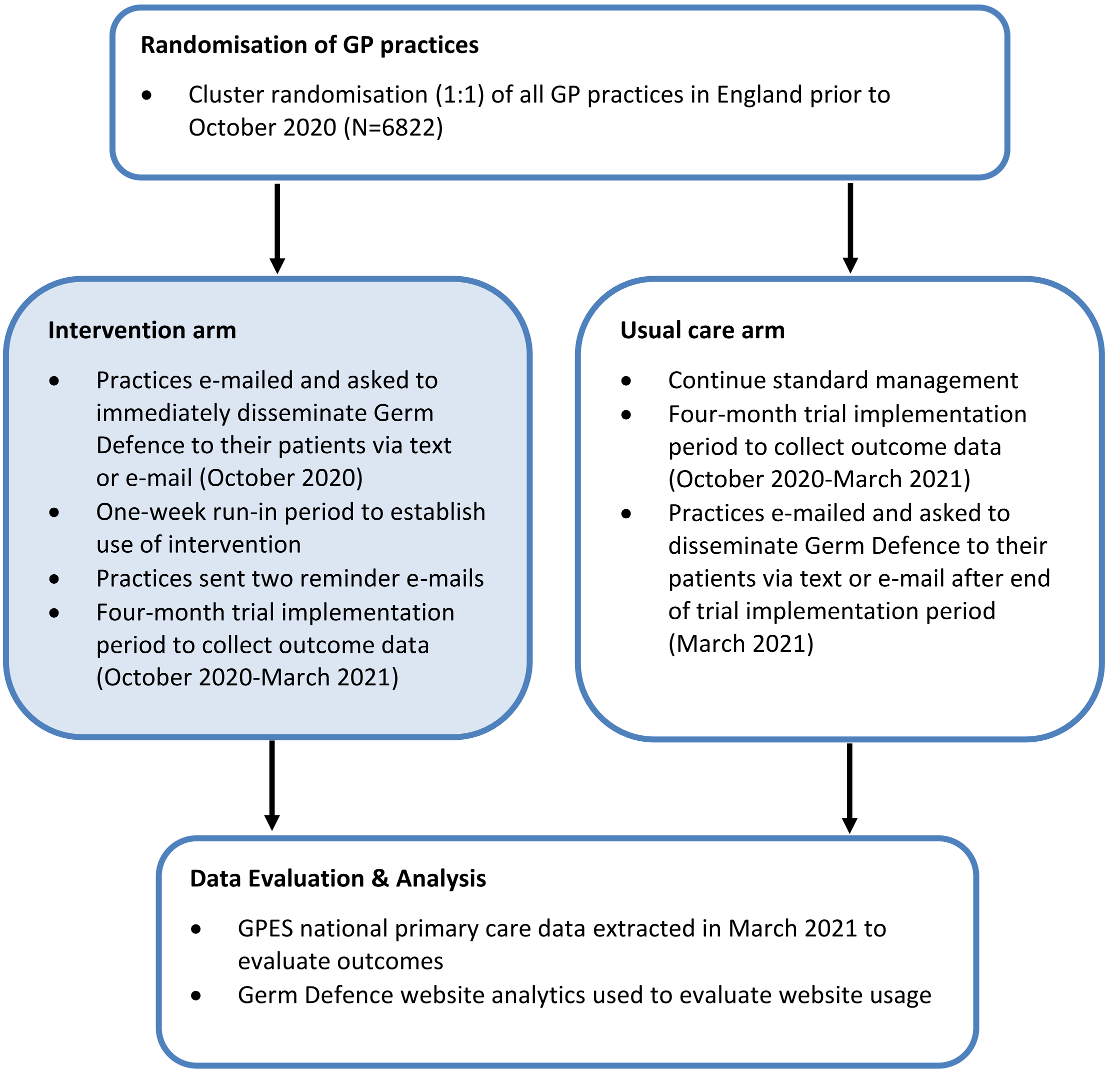

3. RATIONALE

The Germ Defence digital behaviour change website has already proven effective at reducing the spread of viral infections such as colds and flu (Little et al., 2015). It is possible that Germ Defence could also protect people from other respiratory tract infections, including COVID-19, and so increase the capability and capacity of the health service to cope with the number of patients using it. However, even though Germ Defence has been amended to be relevant to COVID-19, we have no evidence that it will be effective. By disseminating the intervention randomly via GP practices to patients, we can examine the effects of implementing Germ Defence in this manner on all respiratory tract infections, including COVID-19 diagnoses.

4. RESEARCH AIM, OBJECTIVES AND OUTCOME MEASURES

4.1 AIM

To examine effects of implementing Germ Defence on respiratory tract infection diagnoses including COVID-19.

4.2 OBJECTIVES

Primary objective
To determine:

1. Whether the Germ Defence intervention decreases the number of respiratory tract infection diagnoses

Secondary objectives
To determine:

1. Whether the Germ Defence intervention results in decreased incidence of COVID-19 diagnoses
2. Whether the Germ Defence intervention results in decreased incidence of COVID-19 symptom presentation
3. Whether the Germ Defence intervention results in decreased incidence of gastrointestinal infections
4. Whether the Germ Defence intervention results in decreased number of primary care consultations
5. Whether the Germ Defence intervention results in decreased antibiotic usage
6. Whether the Germ Defence intervention results in decreased hospital admissions
7. The degree of uptake of GP practices disseminating Germ Defence to their patients
8. Usage of the Germ Defence website by individuals granted access by their GP practice

4.3 OUTCOME MEASURES

These are:

1. Clinical outcomes recorded in routinely collected data in primary care medical records via General Practice Extraction Service (GPES) data for pandemic planning and research (COVID-19) and are routinely collected in anonymised form by NHS Digital or OpenSAFELY Oxford University's secure analytics platform (https://opensafely.org/).
2. Practice engagement in Germ Defence dissemination. Each practice will receive a personalised link to Germ Defence website, enabling monitoring of whether patients from individual practices used the intervention.
3. Germ Defence website analytics which provide overall intervention usage including number of engagements with key behavioural components, average time spent on website, number of pages viewed, perceptions of website usefulness, current and intended behavioural scores.

4.4 MEASUREMENT OF CLINICAL OUTCOMES

Clinical outcomes will be measured using routinely collected data in primary care medical records via the General Practice Extraction Service (GPES) for pandemic planning and research (COVID-19), and from NHS Digital or OpenSAFELY Oxford University's secure analytics platform (https://opensafely.org/) (see section 5.5 about routinely collected data).

5. STUDY DESIGN & SETTING

5.1 STUDY DESIGN

The study is an efficient pragmatic two-arm (intervention v. usual care) cluster randomised trial to reduce respiratory tract infection (RTI) with randomisation at the GP practice level, using routinely-collected patient record data. This design was chosen because Germ Defence is a whole GP practice intervention and does not focus on the actions or outcomes of individual practitioners or patients.

5.2 INTERVENTION

Germ Defence content was developed using theoretical modelling and qualitative research (Yardley et al. 2011), in line with the person-based approach (Yardley et al., 2015.) It draws principally on the theory of planned behaviour (Ajzen, 1985), Leventhal's common-sense model of illness (Leventhal, Phillips & Burns, 2016) and protection motivation theory (Rippetoe & Rogers, 1987). The content, design and structure of the intervention were optimised iteratively using in-depth qualitative 'think-aloud' interviews with members of the general public in order to ensure it is credible, accessible and motivating for as many people as possible (Yardley et al., 2015). Based on process evaluations of the original randomised controlled trial (Little et al., 2015) and previous public dissemination activities (Ainsworth et al., 2017), Germ Defence has been updated and streamlined for use since the coronavirus pandemic, including translation into 20 languages and broadening the infection control behaviours that were recommended. The intervention is a single session, designed to be easily accessible with no sign-up or password required, and the consent process placed within the website privacy policy. Data collection is unobtrusive and kept to a minimum to reduce dropout.

Germ Defence seeks to increase users' perceived risk by emphasising the personal and social health consequences of contracting RTIs including COVID-19. These are followed by messages to increase skills and confidence to reduce exposure to the virus. The Germ Defence content is tailored such that a user selects one of four streams that is relevant to the user's situation:

1: to protect themselves generally.
2: to protect others if the user was showing symptoms.
3: to protect themselves if household member(s) showed symptoms; or
4: to protect a household member who is at high risk.

Content is tailored in this way to encourage users to adopt behaviours appropriate to the perceived level and pattern of risk in their household. Detailed advice is then provided for self-isolating, social distancing, disinfecting and/or cleaning, wearing face-coverings, and putting items aside that may have viruses on them such as shopping/packages, to the extent that users feel is appropriate for the perceived risk. These pages also contain ideas and information on how to structure homes and engage in behaviours safely. The website can be accessed for free at: https://Germ Defence.org/.

5.3 STUDY SETTING

We will contact all GP practices in England, ensuring that the study is rolled out across demographically and geographically diverse regions. We will encourage health care professionals at each intervention practice, to inform their patients aged 16 and over about the Germ Defence intervention. 50% of practices will be contacted and asked to disseminate Germ Defence to their adult patients in Autumn 2020 and 50% will be contacted and asked to disseminate Germ Defence in March 2021.

5.4 ELIGIBILITY CRITERIA

5.4.1 GP Practice inclusion criteria - All GP practices in England

5.4.2 GP Practice exclusion criteria - None

5.4.3 Subject population

We will be collecting anonymised data on adult patients (i.e. those aged 16 and over) who respond to the invitation to look at Germ Defence and this will be gathered using current NHS routine systems. We will not be seeking individual participation or individual consent.

5.5 ROUTINELY COLLECTED DATA

In response to the COVID-19 pandemic, and an increase in demand for primary care data, NHS Digital has leveraged the existing General Practice Extraction Service (GPES) to run a fortnightly data extract from all General Practices in England into NHS Digital to support planning and research. The legal basis for the extract is the COVID-19 Public Health Directions and Control of Patient Information Regulations. GPES data for pandemic planning and research includes patients' demographic and coded clinical and prescription information and can be linked to other datasets including Hospital Episode Statistics (HES), COVID-19 Hospitalisation in England Surveillance System (CHESS) and Office of National Statistics (ONS) mortality data. If GPES data is not available in time we will use OpenSAFELY Oxford University's secure analytics platform (https://opensafely.org/)

5.6 STUDY DURATION

The core elements of the trial will take 7 months to complete - from its launch in October 2020 to its write-up in April 2021. A further 14 months' work will be required to refine the protocol, set-up the study, disseminate its findings and archive study data/documentation. The timings of key tasks can be summarised as follows:

Milestone/task	Start date	Finish date
Protocol refinement and user engagement	April 2020	August 2020
Ethical review and research governance approval	July 2020	September 2020
Randomisation of GP practices	October 2020	October 2020
Germ Defence dissemination - intervention arm	October 2020	-
Follow-up	October 2020	February 2021
Germ Defence dissemination - usual care arm	March 2021	-
Data extraction and cleaning	March 2021	March 2021
Data analysis	March 2021	April 2021
Report writing	April 2021	April 2021
Dissemination of findings	May 2021	November 2021
Archiving of study materials	November 2021	December 2021

The study timetable is plotted as a Gantt chart in Appendix 1: study project plan.

6. RECRUITMENT AND RANDOMISATION

6.1 RECRUITMENT

Study information will be disseminated to GP practices via e-mail. Details of General Practice e-mail addresses will be obtained from publicly available databases or via Clinical Commissioning Groups (CCGs), Clinical Research Networks (CRNs) and the national network of NIHR Applied Research Collaborations (ARCs). The study team will e-mail all practices randomised to the intervention arm asking them to inform all adult patients registered at their practice about the Germ Defence website, e.g. via text or email. Each GP practice will be contacted up to three times during the study, to ensure maximum engagement with the intervention. Each GP practice will be given a personalised website link to send to their patients. This will be used to confirm whether patients have accessed the link through their practice (and therefore whether practices have disseminated the intervention).

The Health Research Authority (HRA) have agreed that since practices will be acting under their own controllership (because we are not dictating how they will inform their patients of Germ Defence) and the study is in the clinical interests of their patients, a Participant Identification Centre (PIC) or Organisation Information Document (OID) will not be needed to comply with regulatory terms.

6.2 RANDOMISATION

Practices will be randomised on a 1:1 basis by the independent Bristol Randomised Trials Collaboration (BRTC) unit. CCGs in England will be divided into blocks according to region, and equal numbers in each block will be randomly allocated to intervention or usual care. The randomisation schedule will be generated in Stata statistical software by a statistician not otherwise involved in the enrolment of general practices into the study. We will ask staff at GP practices randomised to the intervention arm to share the link to Germ Defence with all adult patients registered at their practice during the 4-month trial implementation period and care will otherwise follow current standard management. Patients at GP practices randomised to the usual care arm will receive current standard management for the trial period after which they will be given a link to the Germ Defence intervention by their practices.

7. STATISTICS AND DATA ANALYSIS

7.1 POWER CALCULATION

With an infection rate of 15% in the usual care arm and 14.4% in the intervention arm, we could detect a risk ratio of 0.96. With 90% power and alpha 0.05, we would require 73,195 participants per study arm. Based on an ICC of 0.01 and approximately 7,500 adult patients per practice, we have a design effect of 75.99. With this increase to account for clustering, we will need 11,124,176 participants from approximately 1,484 practices. If we assume that only 25% of the practices will disseminate the information as asked, then we will require 5,936 practices to participate in the study. This assumes that we are increasing the number of both intervention and usual care practices but - given the limited workload required of usual care practices - this should be acceptable. According to NHS Digital, there are currently approximately 6,822 practices across 135 Clinical Commissioning Groups (CCGs) in England and, if all are included, this will give over 90% power for the comparisons. In the previous trial, Germ Defence demonstrated it can help reduce the risk of infection by around 14% (Little et al., 2015).

The relative risk used in this calculation represents the minimum that was thought to be clinically meaningful assuming that social distancing may reduce transmission and, so, lead to a smaller effect than in previous studies. Much is unknown about the likely infection prevalence, the impact of social distancing and how outcomes may cluster by practice. There may be a risk that the use of all practices results in the study being overpowered if the true values of the parameters differ from those estimated and used in the calculation. As a result, results will be interpreted with due caution and with reference to their clinical implications.

7.2 STATISTICAL ANALYSIS PLAN

Summary of baseline data

This cluster randomised controlled trial will be analysed at the practice level. Randomisation will be carried out at practice level and we will not have any direct feedback on whether practices distributed the Germ Defence information to all, some or potentially no patients (and likewise whether individual patients were offered the information and made use of it). We will, therefore, conduct all analyses using aggregated data at the practice level, and consider each practice as a unit for the purpose of analysis. Outcome and covariate data will be aggregated in weekly time-series prior to analysis covering the period from 4 months prior to randomisation until 4 months after randomisation to achieve a minimum of 15% infection rate. An indicator variable will indicate whether data items relate to a time pre-randomisation (0) or post-randomisation i.e. the intervention period (1).

Primary outcome analyses

Standard Intention-To-Treat (ITT) analyses will be the primary outcome analyses. Data will be aggregated for the 4-month post-randomisation period and differences between rates will be directly compared between intervention and usual care practices. Since we will be directly randomising 5,936+ practices we expect there to be no systematic differences between the intervention and usual care, groups and outcomes will be directly compared using weighted t-tests for proportions with the cluster sizes as the weights (Campbell et al., 2000).

Secondary outcome analyses

To obtain more insights in the temporal patterns of implementation we will conduct additional time-series analyses. Data will be analysed using Generalised Linear Mixed Effects Models (GLMM) in a Difference-in-Differences (DiD) design (Beard et al., 2019). A Poisson link function or, in the case of over-dispersion, a negative binomial link function will be used to model the primary outcome i.e. weekly number of respiratory tract infection diagnoses in each practice. Covariates in the models will include seasonal effects, size of practice, level of disease at point of randomisation, and practice level indicators such as area-level socio-economic status, age and sex distributions, week number and the indicator variable. Over the duration of the study we do not expect time-varying confounding by these factors to be an important contributor, nor do we expect missing data to be an issue, so we will directly adjust for these factors in the models instead of assigning propensity score weightings. The intervention effect will be estimated by the interaction between the indicator variable and time.

Process analyses

The Germ Defence website will record usage from the unique identifying website links sent by each practice and will be used to examine whether intervention engagement (i.e. a practice effectively communicating link to patients) predicts i) estimates of intervention effectiveness and ii) key outcome variables. A range of additional behavioural mechanisms, overall patterns of practice and user engagement will also be described using website analytics such as number of users per practice, average time spent on the Germ Defence website or pages visited. This description will be further supported by triangulation with qualitative process analysis data from the main Germ Defence study.

8. DATA MANAGEMENT

8.1 GENERAL APPROACH

As described previously in section 5.5, project data will not be collected directly from individual patients but from a variety of publicly available data sources. A request will be made to the Data Access Request Service (DARS) for GPES data for pandemic planning and research following the procedure outlined Data will be shared by NHS Digital through the Trusted Researcher Environment. A Standard Operating Procedure (v1.1) has been developed by NIHR Applied Research Collaboration West (ARC West) for all Data Transfers related to quantitative data for analysis in support of the Covid-19 response. The data will be transferred securely and encrypted to the research team via the University of Bristol's Facility for the Upload of Large Files (FLUFF) service. A link will be sent to NHS Digital via an encrypted email which will be open for 7 days (if file upload is not completed in this timeframe another link will be generated and similarly shared). The data files will be downloaded upon receipt of an e-mail from NHS Digital that the data has been transferred, and then stored in a dedicated University of Bristol Research Data Storage Facility (RDSF) folder and only the ARC West researchers responsible for the quantitative analysis will have access to the folder which is located on a secure password-protected University network file store-space where access is controlled by use of user accounts and file access control lists. Host servers are located in secure data centres within the University of Bristol estate and are protected by secure automatic locking doors, requiring appropriate University Card (MiFare2) and biometric second factor-controlled access to enter (for limited authorised personnel only) as well as being monitored on CCTV by University security services. Locations of routers and switches are physically restricted to IT Services staff. Once the file has been unencrypted and stored in the RDSF folder, the encrypted file will be deleted.

All data will be collected and retained in accordance with the General Data Protection Regulation 2018 (GDPR) as well as The University of Bristol's Research Data Service 'Guidance on the Retention of Research Records and Data For studies involving human participants, their tissue and/or human data' (version 3.0 September 2019).

No personal data will be collected in this study. Routinely collected data will be entered onto a secure, purpose-built study database, with a series of data validation and cleaning processes carried out throughout the trial. Standard operating procedures (SOPs) for database use, data validation and data cleaning will be available and regularly maintained. Access to the database will be password-protected and restricted to University of Bristol personnel qualified by appropriate experience and training. All data will be securely archived in the RDSF and retained for a minimum of 5 years in accordance with the University of Bristol's Records Management and Retention Policy (IGP-03).

8.2 ARCHIVING

This trial will be sponsored by University of Bristol, and the data custodian will be the Chief Investigator (CI) or someone nominated from the research team. All anonymised research data will be kept indefinitely in line with RCUK policy on open access. Essential study documentation will be retained in a secure location during the conduct of the study and for 5 years after the end of the study in line with University archiving policy, after which time these essential documents will be destroyed. Non-essential study documentation will be deleted at the end of the study.

8.3 DATA SHARING

Data will not be made available for sharing until after publication of the main results of the study. Thereafter, anonymised data will be made available for secondary research, conditional on assurance from the secondary researcher that the proposed use of the data is compliant with the Medical Research Council (MRC) Policy on Data Preservation and Sharing regarding scientific quality, ethical requirements and value for money. A minimum requirement with respect to scientific quality will be a publicly available pre-specified protocol describing the purpose, methods and analysis of the secondary research, e.g. a protocol for a Cochrane systematic review.

9. ETHICAL AND REGULATORY CONSIDERATIONS

9.1 RESEARCH APPROVALS

We believe the proposed research does not raise any untoward ethical issues. Health Research Authority (HRA) approval will be sought for the trial and the protocol and other essential study documents will be reviewed by a UK Research Ethics Committee (REC).

9.2 RISKS AND BENEFITS

Since no individual patients are participating in the trial and the study seeks to assess clinical outcomes from routinely collected NHS data, the risk of harm is minimal. Whilst staff at GP practices will be encouraged to promote the Germ Defence website and the information it contains to all adult patients registered at their practices; the study requires no deviation from usual care.

9.3 STUDY SPONSORSHIP

The University of Bristol will act as Sponsor for the study and will be assigned any delegated responsibilities.

9.4 INVESTIGATORS' RESPONSIBILITIES

The Chief Investigator (CI) and research team will obtain the appropriate research approvals and ensure that any contractual agreements have been signed off by all parties prior to the start of the trial. They will ensure compliance to the protocol and carry out the research in accordance with the UK Policy Framework for Health and Social Care. Any amendments to trial documents will be approved by the Sponsor prior to submission to the REC and the CI will ensure that the research team comply with any amendments made. A Trial Master File (TMF) will be set-up and maintained and any study-specific training requirements will be identified to ensure that the rights, safety and wellbeing of research participants are protected, and that research data are reliable. All members of the Germ Defence research team will be suitably qualified by education, training or experience.

9.5 CONSENT

All data analysed in this study will be extracted in anonymised form from publicly available datasets. No personal data will be collected or retained. As a result, we do not need to secure individual patient consent. Practices which support the study by promoting the Germ Defence website will, in doing so, have been deemed to have given implicit consent to participate.

9.6 CONFIDENTIALITY

The Chief Investigator and the research team will preserve the confidentiality of the practices and patients who participate in this study in accordance with the Data Protection Act (DPA) 2018 and will handle research data according to the principles of the DPA and University of Bristol data protection policies. Data will be securely stored on a password-protected computer at the University of Bristol and will be appropriately backed-up using the latest technology and systems.

9.7 PUBLIC AND PATIENT INVOLVEMENT

Public and patient involvement is a key part of the ongoing dissemination, optimisation and evaluation of Germ Defence. Two public contributors were co-investigators on the original parent study, contributing to the writing of the funding proposal, updating and optimising the content of the intervention, dissemination activities and co-authoring papers. One of these contributors - Cathy Rice - is providing input to this project e.g. by commenting on the trial design and protocol development. She will also be a member of the monthly Project Management Group and so have an opportunity to discuss and guide study progress.

9.8 INDEMNITY

The University of Bristol has arranged Public Liability insurance to cover the legal liability of the University as Research Sponsor in the eventuality of harm to a research participant arising from management of the research by the University. Standard NHS insurance and indemnity arrangements also apply.

10. DISSEMINATION

A comprehensive plan for disseminating the study results will be developed by the Project Management Group (PMG) as part of their monthly meetings.

In order to influence policy and inform healthcare practice, we will collaborate with University of Bristol's Policy Bristol team and use our links with NHS England, the Royal College of General Practitioners (RCGP) and Public Health England (PHE) to disseminate our findings. We will write a brief and constructive report about the main policy implications which we will post on the NIHR ARC West and University of Bristol websites and will also disseminate through social media.

Findings will be presented at primary care and behaviour change conferences (e.g. those of the Society of Academic Primary Care (SAPC), the Royal College of General Practitioners (RCGP) and the UK Society for Behavioural Medicine (UKSBM).

Peer-reviewed journal papers will be produced reporting the results and submitted to high impact journals e.g. British Medical Journal (BMJ), British Journal of Public Health (BJPH) and British Journal of General Practice (BJGP).

We will also consider writing blog posts, for example for GPOnline, Commissioning Elf and The Conversation. A plain language summary of the findings will be made available to study participants and members of the public via the Germ Defence website, GP practices and social media.

11. REFERENCES

Ainsworth, B., Steele, M., Stuart, B., Joseph, J., Miller, S., Morrison, L., Little, P., Yardley, L. (2017). Using an analysis of behavior change to inform effective digital intervention design: how did the PRIMIT website change hand hygiene behavior across 8993 users? Annals of Behavioral Medicine, 51(3), 423-431.

Ajzen, I. (1985). From intentions to actions: A theory of planned behavior. In K. Beckmann (Ed.), Action control (pp. 11-39). Berlin, Heidelberg: Springer.

Beard, E., Marsden, J., Brown, J., Tombor, I., Stapleton, J., Michie, S., West, R. (2019). Understanding and using time-series analysis in addiction research. Addiction. Oct; 114(10):1866-1884 doi: 10.1111/add.14643. Epub 2019 Jul 9.

Bradbury, K., Morton, K., Band, R., Van Woezik, A., Grist, R., McManus R.J., Little, P., Yardley, L. (2018). Using the Person-Based Approach to optimise a digital intervention for the management of hypertension. PLoS One, 13(5), e0196868. doi: 10.1371/journal.pone.0196868

Campbell, M., Mollison, J., Steen, N., Grimshaw, J., Eccles, M. (2000) Analysis of cluster randomized trials in primary care: a practical approach. Family Practice. April 2000, 17(2), 192-196. doi:10.1093/fampra/17.2.192

Evans, R.E., Craig, P., Hoddinott, P., Littlecott, H., Moore, L., Murphy, S., O'Cathain, A., Pfadenhauer, L., Rehfuess, E, Seagrott, J., Moore, G. (2019). When and how do 'effective' interventions need to be adapted and/or re-evaluated in new contexts? The need for guidance. J Epidemiol Community Health, 73(6), 481-482. doi: 10.1136/jech-2018-210840

Leventhal H, Phillips LA, Burns E. The Common-Sense Model of Self-Regulation (CSM): a dynamic framework for understanding illness self-management. Journal of Behavioral Medicine. 2016;39(6):935–946

Little, P., Stuart, B., Hobbs, F., Moore, M., Barnett, J., Popoola, D., Middleton, K., Kelly, J., Mullee, M., Raftery, J., Guiging, Y., Carman, W., Fleming, D., Stokes-Lampard, H., Williamson, I., Joseph, J., Miller, S., Yardley, L. (2015). An internet-delivered handwashing intervention to modify influenza-like illness and respiratory infection transmission (PRIMIT): a primary care randomised trial. The Lancet, 386(10004), 1631-1639.

Little P., Read R., Amlôt R., Chadborn T., Rice C., Bostock J., Yardley L. (2020). BMJ, 369:m1728 doi: 10.1136/bmj.m1728

Rippetoe PA, Rogers RW. Effects of components of protection-motivation theory on adaptive and maladaptive coping with a health threat. J Pers Soc Psychol. 1987 Mar;52(3):596–604

Yardley L, Miller S, Teasdale E, Little P, (2011) Using mixed methods to design a web-based behavioural intervention to reduce transmission of colds and flu. J Health Psychol. 16(2):353–64

Yardley, L., Morrison, L., Bradbury, K., & Muller, I. (2015). The person-based approach to intervention development: application to digital health-related behavior change interventions. Journal of Medical Internet Research, 17(1), e30. doi: 10.2196/jmir.4055

APPENDIX 1: STUDY PROJECT PLAN

Covid-19 Germ Defence Implementation: Timetable

NIHR ARC West in collaboration with Public Health England and the Universities of Bristol, Bath, Southampton, and the West of England

Principal Investigator: Dr Jeremy Horwood
Trial Manager: Dr Melanie Chalder

Study duration: 21 months

Trial start date: 1 October 2020
Trial end date: 30 April 2021

<table>
<tr><th></th><th>April</th><th>May</th><th>June</th><th>July</th><th>August</th><th>September</th><th>October</th><th>November</th><th>December</th><th>January</th><th>February</th><th>March</th><th>April</th><th>May</th><th>June</th><th>July</th><th>August</th><th>September</th><th>October</th><th>November</th><th>December</th></tr>
<tr><td>Ethics review & research governance approval</td><td colspan="6">study set-up</td><td></td><td></td><td></td><td></td><td></td><td></td><td></td><td></td><td></td><td></td><td></td><td></td><td></td><td></td><td></td></tr>
<tr><td>Randomisation of GP practices</td><td></td><td></td><td></td><td></td><td></td><td></td><td>trial</td><td></td><td></td><td></td><td></td><td></td><td></td><td></td><td></td><td></td><td></td><td></td><td></td><td></td><td></td></tr>
<tr><td>Implemention of Germ Defence in GP practices - intervention arm</td><td></td><td></td><td></td><td></td><td></td><td></td><td colspan="5">trial</td><td></td><td></td><td></td><td></td><td></td><td></td><td></td><td></td><td></td><td></td></tr>
<tr><td>Implemention of Germ Defence in GP practices - usual care arm</td><td></td><td></td><td></td><td></td><td></td><td></td><td></td><td></td><td></td><td></td><td></td><td>trial</td><td></td><td></td><td></td><td></td><td></td><td></td><td></td><td></td><td></td></tr>
<tr><td>Data extraction and cleaning</td><td></td><td></td><td></td><td></td><td></td><td></td><td></td><td></td><td></td><td></td><td></td><td>trial</td><td></td><td></td><td></td><td></td><td></td><td></td><td></td><td></td><td></td></tr>
<tr><td>Data analysis</td><td></td><td></td><td></td><td></td><td></td><td></td><td></td><td></td><td></td><td></td><td></td><td colspan="2">trial</td><td></td><td></td><td></td><td></td><td></td><td></td><td></td><td></td></tr>
<tr><td>Report writing</td><td></td><td></td><td></td><td></td><td></td><td></td><td></td><td></td><td></td><td></td><td></td><td></td><td>trial</td><td></td><td></td><td></td><td></td><td></td><td></td><td></td><td></td></tr>
<tr><td>Dissemination of findings</td><td></td><td></td><td></td><td></td><td></td><td></td><td></td><td></td><td></td><td></td><td></td><td></td><td></td><td colspan="7">study closedown</td><td></td></tr>
<tr><td>Archiving of study materials</td><td></td><td></td><td></td><td></td><td></td><td></td><td></td><td></td><td></td><td></td><td></td><td></td><td></td><td></td><td></td><td></td><td></td><td></td><td></td><td colspan="2">study closedown</td></tr>
</table>
